# Unexpected consequences: women’s experiences of a self-hypnosis intervention to help with pain relief during labour

**DOI:** 10.1186/s12884-015-0659-0

**Published:** 2015-09-25

**Authors:** Kenneth Finlayson, Soo Downe, Susan Hinder, Helen Carr, Helen Spiby, Peter Whorwell

**Affiliations:** Research in Childbirth and Health Unit (REACH), School of Health, University of Central Lancashire, Preston, UK; RaFT Research, Lower Hall, Main Street, Downham, Clitheroe, Lancashire UK; Royal Bolton Hospital, Minerva Road, Farnworth, Bolton, Lancashire UK; Faculty of Medicine & Health Sciences, University of Nottingham, Nottingham, UK; Centre for Gastrointestinal Sciences, University Hospital of South Manchester NHS Foundation Trust, Manchester, UK

**Keywords:** Self-hypnosis, Pain relief, Labour, Childbirth, Women

## Abstract

**Background:**

Self-hypnosis is becoming increasingly popular as a means of labour pain management. Previous studies have produced mixed results. There are very few data on women’s views and experiences of using hypnosis in this context. As part of a randomized controlled trial of self-hypnosis for intra-partum pain relief (the SHIP Trial) we conducted qualitative interviews with women randomized to the intervention arm to explore their views and experiences of using self-hypnosis during labour and birth.

**Methods:**

Participants were randomly selected from the intervention arm of the study, which consisted of two antenatal self-hypnosis training sessions and a supporting CD that women were encouraged to listen to daily from 32 weeks gestation until the birth of their baby. Those who consented were interviewed in their own homes 8–12 weeks after birth. Following transcription, the interviews were analysed iteratively and emerging concepts were discussed amongst the authors to generate organizing themes. These were then used to develop a principal organizing metaphor or global theme, in a process known as thematic networks analysis.

**Results:**

Of the 343 women in the intervention group, 48 were invited to interview, and 16 were interviewed over a 12 month period from February 2012 to January 2013.

Coding of the data and subsequent analysis revealed a global theme of *‘unexpected consequences’*, supported by 5 organising themes, ‘*calmness in a climate of fear’, ‘from sceptic to believer’, ‘finding my space’, ‘delays and disappointments’* and *‘personal preferences’.* Most respondents reported positive experiences of self-hypnosis and highlighted feelings of calmness, confidence and empowerment. They found the intervention to be beneficial and used a range of novel strategies to personalize their self-hypnosis practice. Occasionally women reported feeling frustrated or disappointed when their relaxed state was misinterpreted by midwives on admission or when their labour and birth experiences did not match their expectations.

**Conclusion:**

The women in this study generally appreciated antenatal self-hypnosis training and found it to be beneficial during labour and birth. The state of focused relaxation experienced by women using the technique needs to be recognized by providers if the intervention is to be implemented into the maternity service.

## Background

The experience of labour pain is highly variable [1, 2]. Although studies consistently show that many women would prefer to labour without pharmacological pain relief, the percentage of labouring women who actually receive medication for childbirth is rising steadily [3, 4]. However, the link between effective pain relief and maternal satisfaction with labour is not straightforward. Women who have inadequate pain relief during labour are at increased risk of post-traumatic stress in the postnatal period [5] but those who use epidural analgesia are, overall, less satisfied with their experience of labour and birth compared to those who do not [2].

A range of alternative pain relieving solutions has been proposed for labour including acupuncture, immersion in water and hypnosis. Hypnosis, in particular, has been used in maternity care for a number of years with case studies highlighting its benefits as an analgesic dating back to the late nineteenth century [6]. More contemporary studies using a variety of methodologies have produced mixed results. In a large case matched study involving a total of 520 participants across six US states, women receiving antenatal hypnosis training were significantly less likely to need pharmacological analgesia (including epidurals) during labour when compared to controls receiving usual antenatal care [7]. The benefits of hypnosis for pain relief during labour were tentatively supported in a 2010 Cochrane review of complementary and alternative therapies (CAM’s) which found that ‘acupuncture and hypnosis may be beneficial for the management of pain during labour’ [8]. A more specific review of hypnosis for labour and delivery pain, published a year later and including thirteen trials also found evidence to support its use in this context [9]. However, recent findings from two large randomized controlled trials (RCT’s) conducted in Denmark and Australia [10–12], and the most recent UK Self-Hypnosis for Intra-partum Pain (SHIP) trial [13] all found no difference in epidural use between an intervention group receiving self-hypnosis training during the antenatal period and a control group receiving usual antenatal care. Furthermore, in the most recent overview of systematic reviews for labour pain management conducted in 2012 the authors found insufficient evidence to support the use of a variety of CAM’s including hypnosis [1].

Despite the inconsistency of the evidence base, the use of hypnosis in labour appears to be increasing in popularity [14, 15]. Frequent anecdotal accounts of the benefits of self-hypnosis for pregnant and laboring women continue to emerge from practice and the technique remains popular in spite of the current evidence base. Qualitative accounts from women on the use of hypnosis could illuminate areas that are currently being overlooked by quantitative outcomes data. Neither of the two studies of hypnosis for labour that were published immediately prior to the SHIP trial included qualitative data [10, 11] although some of the quantitative findings in Werner’s study suggest that the women in the self-hypnosis group were much more satisfied with their birth experience compared to the controls [16].

Indeed, with the exception of one or two case studies, the only published qualitative research accounts relating to women’s experiences of using self-hypnosis for labour come from a very small Iranian study that included 6 women. The participants reported feeling more confident, less fearful, less anxious and more satisfied with their birth after a course of antenatal hypnosis training [17]. However, the potential transferability of these findings is limited.

Given the lack of evidence in this area, we aimed to explore the views and experiences of a group of women receiving an antenatal self-hypnosis training programme for labour pain relief to inform the results of the SHIP Trial (Downe et al. [13]).

## Methods

### Design

One to one qualitative interviews

### Setting

Interviews were conducted alongside the main SHIP Trial, a randomized controlled trial investigating the effect of an antenatal self-hypnosis training programme on rates of epidural use amongst laboring women. The 678 participants in the trial were randomly assigned to the intervention group (self-hypnosis training; *n* = 343) or the usual care group (*n* = 335). Both groups received the standard package of antenatal education delivered by the NHS Trust and, in addition, intervention participants received group tuition in the use of self-hypnosis for anxiety and pain relief during labour. The tuition consisted of two 1.5 h training sessions at around 32 weeks and 35 weeks pregnancy, supported by a take-home, practice CD that participants were encouraged to listen to on a daily basis from the first training session until the birth of their baby. All of the women (in both groups) were aged 18 or more and were experiencing their first pregnancy. Birth companions/partners were recruited alongside the women in both groups and, where possible, attended the intervention or educational training sessions in a supportive role. Clinical data relating to the outcomes of the trial were collected at the Trust site and additional data were collected from the trial participants via questionnaires distributed at four time points: baseline (27 weeks gestation), 36 weeks (after the intervention) and 2 and 6 weeks post-natal (Downe et al. [13]).

The trial was conducted over a three year period (2010 – 2013) in three NHS Trusts covering six different clinical sites (three consultant unit, two free standing units, one alongside unit) in the North West of England.

### Ethical approval

Ethical approval for the trial, including the qualitative interviews, was obtained from the National Research Ethics Service (NRES) (Study Reference 10/H1011/31) and the University of Central Lancashire Research Ethics Committee, and all relevant governance procedures were approved by the participating Trusts prior to recruitment. The trial was registered on a publically available database in accord with National Institute for Health Research (NIHR) guidelines and given the number ISRCTN27575146.

### Procedures

Participants in the interview phase of the study were recruited over a 12 month period (Feb 2012 – Jan 2013) from the primary NHS site. Four participants from the intervention group were randomly selected each month and contacted by a member of the research team to see if they would be willing to take part. Those who agreed were sent a consent form prior to the interview and, once a signed copy was returned, arrangements were made to interview the participants in a private location of their choosing. Although we expected to recruit around 20 women, the intention was to continue selection of participants until our iterative process of analysis revealed that no new themes were emerging from the data. Where possible, we sought to interview women with their birth partner/ companion, and, in these cases an additional consent form was sent to the birth companion for information and signature.

The interviews were semi-structured in format and included two key questions as well as a series of supplementary, follow up questions. The key questions were, ‘*Can you tell me about your experience of the self-hypnosis training programme?’* and ‘*can you tell me about your labour and birth?’* All interviews were conducted by a member of the research team (KF or HC) and were recorded using a digital device. Each interview took place in a quiet room in the participant’s home and lasted between 35 and 80 min. At the time of the interviews all of the women were between 8 and 14 weeks post-partum. We chose this timeframe because participants were still completing follow-up questionnaires from the SHIP trial (at 2 weeks and 6 weeks post-natal) and we didn’t want to add to their existing trial commitments during this busy period.

## Results

### Participants

During the 12 month data collection period 48 women were randomly selected for interview from the lead Trial site. Of these, 21 did not answer our initial request to take part, 6 declined to be interviewed (usually citing lack of time), four did not complete the self-hypnosis training programme (three of these went into labour prematurely and were unable to attend the second training session) and one did not attend for the interview. A total of 16 women and three birth companions agreed to be interviewed.

The participants were all first time mothers aged between 23 and 38 with an average age of 29.6**.** All of the women gave their ethnic origin as ‘white British’. Although this is unreflective of the general population at the study site, where around 21 % self-identify as non-white (mainly Asian), it is reflective of the trial population where only 6.4 % identified themselves as something other than ‘White British’. All of the women had a birth companion (either husband or partner) who came to the training sessions with them. This was very similar to the intervention group as a whole where 89 % had a birth partner who attended. 12 of the 16 women received midwife-led care, similar to the trial average of 72 %, and the Trust average of 70 %. Three of the 16 (19 %) used epidural analgesia for labour, lower than the trial average of 28 % and the Trust average of 30 %.

### Analysis

For the data analysis we used thematic network analysis, a technique that adopts a less abstract approach to qualitative data analysis and presents the findings in a methodical, transparent and organized manner [18]. Using this method each interview was transcribed by a member of the research team (KF, SD, SH, HC) and, after coding the data, preliminary themes were generated by each author independently. These preliminary themes were then discussed among the team and, using an iterative process, draft basic themes were developed by consensus. Following the review and analysis of 16 transcripts the team felt that no new themes were emerging and data collection was suspended. The draft themes were finalized and discussed by all members of the team and following further consensus discussions the basic themes were amalgamated (collapsed) into broader, more abstract, organizing themes. These were then used to develop a principal organizing metaphor or global theme.

**Fig. 1:**
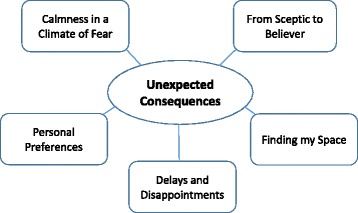
Summary of organising themes and global theme

The findings are represented by five organizing themes: *‘Calmness in a Climate of Fear’, ‘From Sceptic to Believer’, Finding my Space’, ‘Delays and Disappointments’* and *‘Personal Preferences’.* These organizing themes are further encapsulated by the global theme of ‘*Unexpected Consequences’*. These themes are displayed in Fig. 1 and are discussed below.

### Calmness in a climate of fear

All of the women described feeling anxious or fearful about labour and birth before they attended the self-hypnosis training sessions. These feelings were often compounded by hearing negative stories about childbirth in the media as well as from friends and family members. A number of women specifically highlighted the influence of maternity based reality TV programmes like ‘One Born Every Minute’ and recalled how these types of programmes elevated existing levels of fear and anxiety.

*“‘One Born Every Minute’ is a stupid thing to watch, but I was compelled to watch it and I was petrified of losing all dignity and being ridiculous and rude to people and all the rest of it”. [Julie]*

*“I kept watching that ‘One Born Every Minute’ programme and it makes you more frightened”.* [Shirley]

However, after the self-hypnosis sessions women reported feeling calmer and more confident about their upcoming birth.

*“I remember being a lot more worried and then after these sessions [Self-Hypnosis] I wasn’t as worried. Afterwards it was completely different but definitely it changed my thoughts towards it. It made me more…I don’t know…I wasn’t as scared”.* [Katy]

Many of the women recognized that they might still feel scared when they went into labour but would now have something they could use to reduce and control any feelings of anxiety.

*“So I trusted that if I could focus and manage my pain through thinking my way through it, then I had something to use basically….without that I think I would have panicked a lot because I wouldn’t have had anything”* [Julie].

### From sceptic to believer

Although a few of the women indicated that they joined the trial because of a general interest in complementary medicine, the majority of the interviewees felt ambivalent about the intervention, and took part in the trial because it was perceived to be ‘safe’ and ‘wouldn’t do any harm’**.** Some admitted to being more sceptical, and a few even felt dismissive before the training programme began. However, after the first session these rather ambivalent views were often superseded by a much more positive outlook.

*“I went into it thinking ‘this is going to be a load of rubbish’ and I came out of the first session thinking, ‘that could actually work’.* [Debbie]

The more sceptical participants frequently mentioned a specific 15 min section of the first training session as being of particular significance. This component of the training included a very simple description of the physiology of labour and birth and was intended to provide a basic grounding before the self-hypnosis training began. Despite the mass of information many of the participants had already encountered by this stage of their pregnancy, it was striking how many of them found this information new and fascinating:

*“I was a bit sceptical about it [Self-hypnosis] but when I went to the first class and she explained the physiological sort of thinking behind it I thought ‘ooh, it makes sense’….I understood, and I felt a lot more positive about it”.* [Ruth]

*“….she talked about the fight or flight sort of response, and how it was supposed to keep everything calm….the blood flow to the uterus to make it less painful and what have you,… so yeah we felt really positive after that one” [the first session]* [Gemma]

Several women who had initially been quite dismissive of self-hypnosis prior to the training, went on to explain how they had been able to use the technique in other contexts or situations where they felt particularly anxious or fearful, such as going to the dentist, or to help with breastfeeding difficulties.

*“After I had him we were having problems with breastfeeding… I couldn’t sleep because I was that stressed about not being able to feed him, so putting that [CD] on the headphones helped me to relax”.* [Julie]

### Finding my space

This theme captures the variety of ways in which participants expressed their sense of finding space, in both a literal and a metaphorical sense. From a practical perspective women found it a struggle to incorporate the daily routine of listening to the self-hypnosis CD into their already busy lives. Listening to it at work or in a shared living space often proved difficult and, with work, social and pregnancy related schedules to maintain, finding the required 25 min often meant leaving practice until the last thing at night. This meant that they played it to themselves in their bedrooms, sometimes in bed, and invariably fell asleep before the end of the CD.

*“I wouldn’t have had any time before I went to bed to be honest….that was the only time because I was a teacher so you’ve got all your work to do at night time and everything so I did find it quite easy to just stick it on [CD], listen to it and fall asleep and....yeah, it was good for me”.* [Katy]

When women found a way to incorporate the daily practice into their schedule they began to look forward to it and appreciate the associated feelings of relaxation, irrespective of whether they fell asleep or not.

*I’d sort of get ready and think, ‘right, now you’ve been to the toilet and you’ve switched the phone off’, and then its relaxing time and I sort of really enjoyed it….definitely.* [Shirley]

From a hypnotic perspective, the participants described how the daily practice eventually generated an imaginary space or place in their minds that they could visit while in a hypnotic state. This became both a source of strength as well as a place of refuge and was used by some of the women to manage feelings of anxiety and pain during labour.

*I think visualising a place to go to helped, because at the height of the contractions if you’ve nowhere else to go you’re just in that pain….visualising that place helped me with those contractions because my waters broke quite quickly and it was quite intense…. my contractions were very frequent and strong throughout really, so having somewhere to go when another one was coming back, when I hadn’t had a minute in between, was really helpful to me.* [Julie]

### Delays and disappointments

Although all of the participants were largely positive with their comments about the self-hypnosis programme, several women described potential shortcomings when it came to using the techniques they had learned in a clinical setting. This was particularly evident when they arrived at a hospital or birth centre, apparently in active labour, and were not taken seriously by the admissions staff because they were ‘too relaxed’.

*This is a side effect of the self- hypnosis …I mean I may be completely wrong, but the way I read it was, because I wasn’t a gibbering wreck she [the midwife] thought that I wasn’t as bad as I thought I was because I was able to say, ‘yes I am ready, please don’t send me home’, but I wasn’t really upset about things. So, yeah, I do think that was a side effect of being relatively calm.* [Julie: Sent home and returned to be admitted in established labour 45 mins later).

*“I found that frustrating that they’d be like, ‘oh no, you don’t look…you know…sad enough to be in labour’, and I’m like, ‘I can feel the pain, I’m just smiling’, so…I felt like you had to be screaming and shouting. I felt like if you were calm then maybe they didn’t think you were in labour”. [Sandra]* Went on to have an emergency caesarean section

For other participants the reassurance and confidence they had learned during the training programme was shattered when they arrived in the labour ward. Self-hypnosis raised their expectations of the type of birth they could experience and when this did not materialise they were left with feelings of frustration and disappointment.

*“Situations didn’t flow how I expected them to, you know….umm… they say to you when you go to these [Self-hypnosis] sessions, ‘be positive’, ‘it’s all about you’, you know, ‘you’ll be going into the birth suite and it’s all going to happen all naturally and easily’ and it doesn’t always happen like that does it?”* [Caroline] Had induction followed by caesarean section**.**

### Personal preferences

Although the practice CD was exactly the same and the structure and content of the training sessions were standardized to ensure fidelity, participants developed a number of ways to utilize the training resources. Some women transferred the CD content on to a mobile device so they could listen to it in different locations, such as at work, or in a particular setting, like the bedroom, where there was no access to a CD player.

*“I just put it [CD] on my phone, it was much easier and more convenient…I’ve still got it on there”!* [Vicky]

Participants became particularly creative with their choice of listening place and sometimes tried to tie this in with their expected birth location to make the practice more meaningful**.**

*“When I realised I wanted a water birth I started listening to it in the bath”* [Joanne]

Listening to the same voice for 25 min each day for 7–10 weeks gave rise to some comments about the quality and tone of the voice and the urge to change the way they listened to the CD. Some women would ‘get stuck’ on a particular word or phrase, because it amused them or grated on them or was pronounced in a distinct regional accent. These participants suggested it might be better to have the same voice as the training sessions on the CD (the midwife) or to have background music instead of the voice alone.

*“I thought maybe if it had like background music on it to make it a bit more… because everything is so quiet and it’s just a voice…because I think sometimes when you listen to music you’re a bit more relaxed”* [Ruth]

Other participants were keen to make suggestions about the length of the sessions or the timing of them or the number they received. Most felt that two were not enough and that an additional one closer to the delivery date would have been of benefit.

*“…don’t get me wrong there was a lot of information in those two sessions, but I think we probably would’ve benefitted from a few more really”* [Caroline]

### Unexpected consequences

The global theme of ‘unexpected consequences’ emerged from the data at multiple levels. Firstly the women were surprised by the increased sense of calmness and relaxation they experienced after the first training session, and by the applicability of the techniques in a range of stressful situations, even when they had been relatively unconvinced about the value of self-hypnosis when they agreed to enter the trial. Secondly, the research team were surprised to find that the element of the sessions that was most often remembered as useful was the short introduction to labour physiology. Finally, the women’s accounts suggest that the midwives they met when they arrived on the labour ward were unprepared for their relaxed appearance, and, therefore, assumed that their labour was not advanced.

## Discussion

Women’s experiences of self-hypnosis as part of the SHIP trial were generally very positive. Our findings indicate that women arrived at the first self-hypnosis session feeling anxious, fearful and occasionally sceptical and, after completing the training, felt confident, empowered and reassured. This transition is similar to the findings reported in the only other qualitative study of women’s experiences of an antenatal, self-hypnosis training programme, conducted in Iran in 2009 [18]. The authors highlight women’s ‘sense of relief’, ‘increased self-confidence’ and ‘satisfaction’ following their involvement with the hypnosis intervention. They also note that women felt less anxious and less fearful after the hypnosis training – findings reflected in our ‘calmness in a climate of fear’ theme. Interestingly, for the women in our study, the initial reduction in levels of fear and anxiety was attributed to a 15 min section in the first training session where the midwife presented a short explanation of the physiology behind labour and birth. This is an interesting finding as although much has been written about the psychological, supportive and practical components of antenatal education [19, 20], the relevance of women’s understanding of the physiology of labour and birth is less well understood. This relatively simple approach to alleviating anxiety may be significant as there are well established theories propounded over fifty years ago linking raised levels of anxiety and fear during pregnancy with increased levels of pain during labour [21]. Supporting evidence for this theory is increasing and more recent studies have shown that elevated levels of fear or anxiety during pregnancy can lead to increased rates of medical intervention including epidural use [22–25].

It is also likely that reduced levels of anxiety and increased confidence about labour and birth will lead to a more satisfying birth experience. Although the women in these interviews did not discuss their levels of satisfaction directly it is clear that the majority felt happy with their experience and, when prompted, all 16 said they would use the technique again in any future pregnancies. These findings are similar to those from the recent Danish self-hypnosis trial which showed that women receiving the hypnosis intervention were significantly more satisfied with their birth experience compared to a relaxation group and a control group [17]. The same authors, and the SHIP trial data, also note that levels of fear and anxiety in specific circumstances were significantly lower in the hypnosis trained intervention group [11, 14]. This finding is supported by previous trials of this phenomenon [26], [8].

For some women, the ability to control their anxiety and act in a calm and composed manner led to confusion at hospital admission where staff were more used to seeing women arriving in a state of distress. The important role of labour ward staff as gatekeepers has been identified in previous studies of women’s experience of attempting to gain access to intrapartum maternity care [27]. The perception that some staff misinterpreted signs of labour due to the unexpectedly relaxed state women were in when under hypnosis is an important finding. It strongly suggests that any Trust intending to set up antenatal self-hypnosis sessions should ensure that all of the staff women may encounter are aware of the altered behavioural norms for women using self-hypnosis when in active labour.

For other women, an unexpected consequence of using self-hypnosis was that it raised expectations and led to disappointment when labour and birth failed to live up to these enhanced expectations. We were aware of this during the planning stage of the study and tried to address the issue at both of the training sessions by highlighting the unpredictability of labour and birth. However, the hypnosis scripts were deliberately designed to emphasize feelings of confidence, empowerment and relaxation as these are considered essential components of self-hypnosis training in this context. It may be that any form of antenatal intervention that seeks to reduce levels of fear and anxiety will invariably raise expectations, with subsequent disappointment for a sub-set of women who do not meet their revised expectations. Alternatively, this may be a more general issue, independent of how a woman prepares for labour and birth; if women underestimate or downplay the intensity of the pain they expect to encounter during labour this may influence their experience of birth in a negative way [5].

At a methodological level, we were intrigued to note the range of strategies women used to individualize the adoption of self-hypnosis practice into their lives during the antenatal period. This raises questions about how far randomised trials of complex interventions can, or, indeed, should, permit flexibility in the means of delivery of an intervention, when the aim of the study is pragmatic: ie, when the intention is to find out if the intervention would work in the uncontrolled realities of everyday lives. [28, 29]

### Strengths and limitations

Apart from one small study of 6 women undertaken in Iran, this is the only published study we are aware of that explores women’s views of using self-hypnosis in labour. The participants were selected randomly, and are representative of those in the index randomised trial. The procedures used for data collection and analysis were rigorous and transparent, and the data interpretation was reached by consensus among the research team. However, less than half of the women invited to take part did so, and they were all from one specific ethnic group (White British). Women who did not access the training did not take part, and it is possible that those with a less positive experience did not respond to the interview invitation. This should also be viewed in the light of the primary data from the SHIP trial in which, for the whole study population, most outcomes were not affected by the hypnosis training [14]. The contrary findings emerging from the qualitative data could suggest either that interviewed women were unusual in finding the intervention helpful, or that the outcomes chosen for the main study were not those that were most important for women’s sense of well-being.

Although birth partners were invited to the self-hypnosis training and often participated in practice (listening to the CD) we did not interview them directly. Women occasionally referred to the positive contributions of their partners in the interviews but this is not reflected in our data. Findings from a previous study in which birth partners played an active role in prompting self-hypnosis during labour showed that levels of pain were significantly lower in the partner involvement group compared to a control group [30]. We would therefore recommend that the role of the partner in assisting with hypnosis is explored in future qualitative studies in this context. Despite these limitations, this remains the largest study of women’s views and experiences of self-hypnosis training for labour pain to date.

## Conclusion

Women randomized to self-hypnosis training to help with labour pain who agreed to be interviewed about their experiences reported unexpectedly positive experiences during labour and birth. In many cases, initial scepticism was replaced by a confidence and belief in the technique which extended to use in other contexts. Despite leading relatively busy lives these women were able to incorporate regular practice into their daily routine and used a range of novel strategies to personalize the relatively rigid trial protocol to suit their needs. One unanticipated consequence of the deep level of relaxation achieved by women using hypnosis was that midwives did not always recognize labour progress at admission and this sometimes led to delays and frustration. A few participants also noted that the raised expectations associated with the training led to some disappointment when their labour and birth failed to proceed as planned. These findings indicate that the use of self-hypnosis for labour pain was valued and appreciated by the participants in this interview study but the experiences and expectations of both service users and staff need to be considered when decisions are made about the introduction of the technique into labour and birth preparation sessions.

## Consent

All authors agreed that the final submitted version was suitable for publication.
